# Prevalence of other autoimmune diseases in polyglandular autoimmune syndromes type II and III

**DOI:** 10.1007/s40618-020-01229-1

**Published:** 2020-08-17

**Authors:** G. Pham-Dobor, L. Hanák, P. Hegyi, K. Márta, A. Párniczky, M. Gergics, P. Sarlós, B. Erőss, E. Mezősi

**Affiliations:** 1 First Department of Medicine, Medical School, University of Pécs, 13 Ifjúság, Pecs, 7624 Hungary; 2 Szentágothai Research Centre, University of Pécs, Pecs, Hungary; 3 Institute for Translational Medicine, Medical School, University of Pécs, Pecs, Hungary; 4 Heim Pál National Institute of Pediatrics, Budapest, Hungary

**Keywords:** Polyglandular autoimmune syndrome, Addison’s disease, Autoimmune thyroid disease, Diabetes mellitus, Meta-analysis

## Abstract

**Purpose:**

Polyglandular autoimmune syndromes (PAS) are complex, heterogeneous disorders in which various autoimmune diseases can occur, affecting both endocrine and non-endocrine organs. In this meta-analysis, the prevalence of associated autoimmune disorders was investigated in PAS II and III.

**Methods:**

A comprehensive search in MEDLINE and Embase databases identified 479 studies with the keywords of PAS II and PAS III. 18 records containing a total of 1312 patients fulfilled our inclusion criteria (original studies reporting at least 10 cases and containing the combination of other autoimmune disorders) and were selected for further analysis. A meta-analysis of prevalence was performed using the random-effects model with the calculation of 95% confidence intervals (CI). Results of each meta-analysis were displayed graphically using forest plots.

**Results:**

Distinction between PAS II and PAS III was made in 842 cases, of which 177 and 665 were PAS II and III (21.1 vs 78.9%), respectively. The prevalence of Hashimoto’s thyroiditis was significantly higher than that of Graves’s disease (39% [95% CI 17–65%] vs. 4% [95% CI 0–10%], respectively; *p* = 0.001). In PAS II, Addison’s disease (AD) coexisted with AITDs, T1DM or the combination of these conditions in 65, 18 and 10% of cases, respectively. In addition, one other endocrine and five non-endocrine organ-specific autoimmune disorders were reported. In PAS III, two other autoimmune endocrinopathies, six non-endocrine organ-specific, and four systemic autoimmune disorders were found in combination with AITDs.

**Conclusions:**

AITDs, T1DM and AD are the most common combinations in PAS, thus screening for these conditions seems to be reasonable.

**Electronic supplementary material:**

The online version of this article (10.1007/s40618-020-01229-1) contains supplementary material, which is available to authorized users.

## Introduction

Autoimmune polyglandular syndromes (PAS) are complex, heterogeneous disorders in which various autoimmune diseases can occur, affecting both endocrine and non-endocrine organs. The diagnosis of PAS can be established if at least two organs are damaged by the autoimmune process [1]. PAS can be further divided into four subgroups based on the affected organs [2]. PAS I or early-onset PAS develops as a result of the mutation of the autoimmune regulator (AIRE) gene and is characterized by the presence of Addison’s disease (AD), mucocutaneous candidiasis and hypoparathyroidism [2]. PAS II is defined by the presence of AD and autoimmune thyroid diseases (AITDs) and/or type 1 diabetes mellitus (T1DM) [2]. In PAS III, AITD can co-occur with any autoimmune disorder, but not with AD [2–5]. Patients who cannot be included in the previous three subgroups are classified as PAS IV [2–6]. Further classifications can also be found in the literature. Authors Frommer et al. and Kahaly et al. proposed a distinction between juvenile (I) and adult (II–IV) forms of PAS [1, 7]. Their definition of PAS III is also different, as they consider the presence of both AITD and T1DM (without AD) obligatory for this group [1, 7]*.* Most studies analyzed in our paper divided PAS III into several further subgroups, which are presented in detail in Table 1.

**Table 1:** Definitions of polyglandular autoimmune syndrome type II and type III across the studies included

Article	Polyglandular autoimmune syndrome type II	Polyglandular autoimmune syndrome type III
Abrar-Ahmad [29]	AD and AITD and/or T1DM and other autoimmunities	The absence of adrenal insufficiency III/a-AITD and T1DM and sarcoidosis or coeliac disease III/b-AITD and pernicious anemia III/c-AITD and vitiligo or alopecia
Ben-Skowronek et al. [27]	AD and AITD and/or T1DM	AITD plus other endocrinopathy except for AD III/A-AITD and T1DM III/B-AITD and pernicious anemia III/c-AITD and vitiligo and/or alopecia and/or other organ-specific autoimmune diseases
Betterle et al. [31]	AD and AITD and/or T1DM	AITD and other autoimmune diseases excluding AD and/or hypoparathyroidism
Cruz et al. [20]	AD and AITD and/or T1DM	PAS type III differs from type II by the absence of AD
Dittmar and Kahaly [21]	AD and AITD and/or T1DM	Apart from the absence of AD, there is no clinical difference between PAS type II and type III
Horie et al. [23]	^a^	AITD and T1DM
Papadopoulos and Hallengren [25]	AD and AITD and/or T1DM without hypoparathyroidism or chronic mucocutaneous candidiasis	^a^
Papadopoulos et al. [26]	AD and AITD and/or T1DM	AITD and T1DM and/or pernicious anemia and/or vitiligo/alopecia
Piatkowska and Szalecki [22]	^a^	AITD and at least one of autoimmune disease: T1DM, pernicious anemia, vitiligo, alopecia areata, coeliac disease, hypogonadism, myasthenia gravis and others excluding AD
Sastre-Garriga [28]	AD and AITD and/or T1DM for primary criteria, secondary criteria can be gonadal failure, vitiligo, alopecia and pernicious anemia	^a^
Storz et al. [35]	AD and AITD and/or T1DM	AD excluded
Choudhuri et al. [19], Handa and Dogra [34], Karagüzel et al. [32], Karavanaki et al. [33], Kondonouri et al. [26], Renzullo et al. [3], Teufel et al. [30]	^a^	^a^

^a^No definition given in the study, we characterized the subgroups, as follows: polyglandular autoimmune syndrome type II: Addison’s disease and autoimmune thyroid disease and/or type I diabetes mellitus; polyglandular autoimmune syndrome type III: autoimmune thyroid disorders and other autoimmune diseases except Addison’s disease

*AD* Addison’s disease, *AITD* autoimmune thyroid disorders, *PAS* polyglandular autoimmune syndrome, *T1DM* type 1 diabetes mellitus

The typical characteristics of PAS are the lymphocytic infiltration of endocrine and non-endocrine organs, the presence of autoantibodies against the affected organs and the defects of cellular and humoral immune responses [4, 8, 9]. In PAS II and III, the main affected genes are the HLA, CTLA-4 and PTPN-22 genes [10]. Beyond the autoimmune disorders which are obligatory for the diagnosis, both PAS II and III can be associated with a wide variety of other autoimmune conditions. Autoimmune disorders of the endocrine glands such as AITDs, T1DM, AD, premature ovarian failure (POF), hypoparathyroidism or hypophysitis may be combined with non-endocrine organ-specific autoimmune disorders including vitiligo, alopecia, inflammatory bowel diseases (IBD), coeliac disease, autoimmune hepatitis, pernicious anemia, haemolytic anemia, multiple sclerosis or myasthenia gravis. Furthermore, systemic autoimmune disorders such as rheumatoid arthritis (RA), systemic lupus erythematosus (SLE), psoriasis, systemic sclerosis and polymyositis could also be comorbidities [11]. The prevalence of autoimmune disorders has been showing an increasing trend for the last decade [12]. Unfortunately, these disorders are usually considered to be isolated diseases instead of comorbidities, which could be one reason for the low number of publications that includes data from larger patient populations in the literature. Another difficulty is that some review articles present data about potential co-associated disorders without defining them as PAS, i.e. the work of Fallahi et al., who demonstrated that several endocrine, non-endocrine organ-specific and systemic autoimmune diseases are more frequent in AITD patients [13]. It is also important to note that the prevalence and type of co-associated autoimmunities can be remarkably different in pediatric and adult AITD patients. Furthermore, certain autoimmune disorders such as T1DM and juvenile idiopathic arthritis (JIA) often present in childhood, whereas co-associated AITD might appear later during adulthood, altering both the initial diagnosis and the prevalence of PAS in childhood [14].

The aims of this meta-analysis were to collect studies systematically to characterize the prevalence of autoimmune diseases other than the obligatory manifestations of PAS II and PAS III and to investigate the characteristics of PAS II and PAS III in relation to gender and age. It is not clear yet which comorbidities can appear with AD, therefore we also aimed to identify both the autoimmune diseases which co-occur with AD and those disorders which do not. Here, we present a systematic review and the first meta-analysis to verify the combinations of autoimmune disorders in PAS II and III.

## Methods

This meta-analysis was reported in accordance with the recommendations of the Preferred Reporting Items for Systemic Reviews and Meta-Analyses (PRISMA) statement [15] and was registered in PROSPERO with registration number CRD42019126826.

### Search strategy

We searched the following electronic bibliographic databases: MEDLINE (via PubMed) and EMBASE, and used only ‘human’ filter. There was no language restriction. The search was completed by 5th November 2018, with the following keywords: “autoimmune polyglandular syndrome”, “autoimmune polyendocrinopathies”, “autoimmune polyglandular syndrome type II” and “autoimmune polyglandular syndrome type III”. The EndNote software (version: X 7.0.2., Clarivate Analytics, Philadelphia, PA, USA) was used to manage all references to remove duplicates and facilitate the selection process.

### Inclusion and exclusion criteria

For inclusion, publications had to demonstrate that (1) the study focused on patients with two or more autoimmune disorders of endocrine and non-endocrine organs and (2) the study showed data of at least 10 cases. Publications were excluded (1) in the case of overlapping study populations and (2) if the study focused on patients only with autoimmune disorders of non-endocrine organs. Review articles were also excluded. The records were independently screened by two investigators against the inclusion and exclusion criteria.

### Data extraction

A standardized form of numeric data was independently extracted by two investigators and manually populated into a purpose-designed Excel 2016 sheet (Office 365, Microsoft, Redmond, WA, USA) containing the following information: year of publication, type of study, participating centers, number of patients included and diagnosed with PAS, average age of the patients, sex, main disorders (AD, AITDs, T1DM, POF, vitiligo, alopecia, coeliac disease, autoimmune hepatitis, pernicious anemia, autoimmune bowel disease, haemolytic anemia, SLE, psoriasis, Sjögren’s syndrome, RA, myasthenia gravis, multiple sclerosis, hypoparathyroidism, hypophysitis) and their combinations. The authors of the original studies were contacted to obtain missing information.

### Statistical analysis

The methods recommended by the working group of the Cochrane Collaboration [16] were used for the synthesis of data. Event rates (in percentage distribution) were calculated for dichotomous outcomes. Random-effects model was applied for all analysis with the DerSimonian–Laird estimation [17]. Statistical heterogeneity was analyzed using the *I*^2^ and the *χ*^2^ tests to gain probability values; *p* < 0.1 was defined to indicate significant heterogeneity. Statistical analyses were performed with Stata 15 (Stata Corporation, College Station, TX, USA).

### Assessment of the quality of the included studies

Two review authors independently assessed the quality of the included studies as recommended by Murad et al. [18] (Supplementary Table 1). Disagreements between the review authors over the quality were resolved by discussion, with the involvement of a third review author where it was necessary.

## Results

### Characteristics of the studies included

**Fig. 1:**
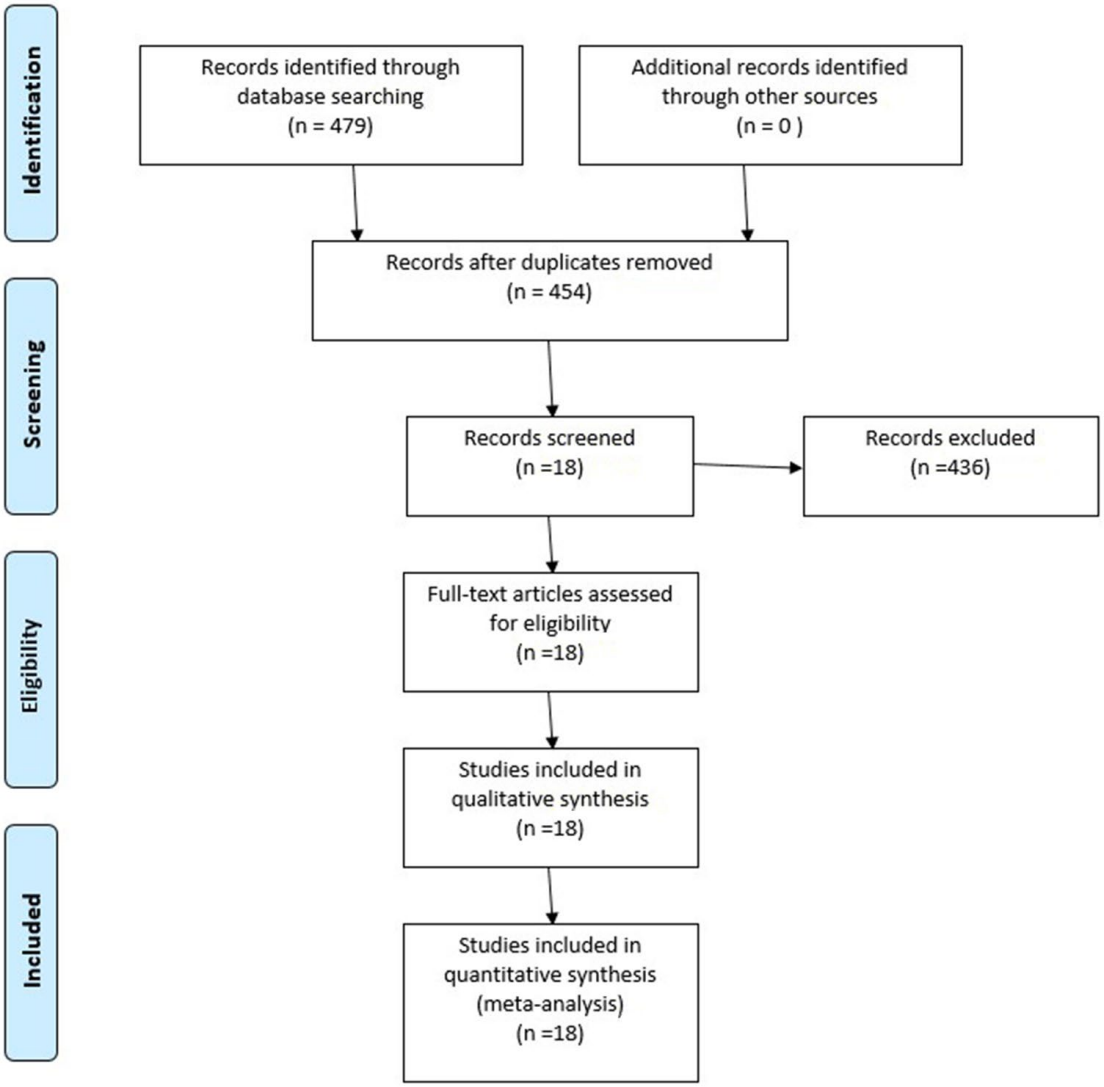
PRISMA flow diagram

After the comprehensive search in MEDLINE and Embase, 479 records were identified. After removing duplicates, we reviewed the remaining 454 records against the eligibility criteria, and excluding case reports, 18 articles containing a total of 1312 patients (10–254/papers) were selected for further analysis. Our PRISMA flow chart of the selection process is shown in Fig. 1. Caucasian, Asian and Hispanic ethnicities were included. Characteristics of the studies included in PAS II and III are summarized in Supplementary table 2.

### Characteristics of the patients

**Fig. 2:**
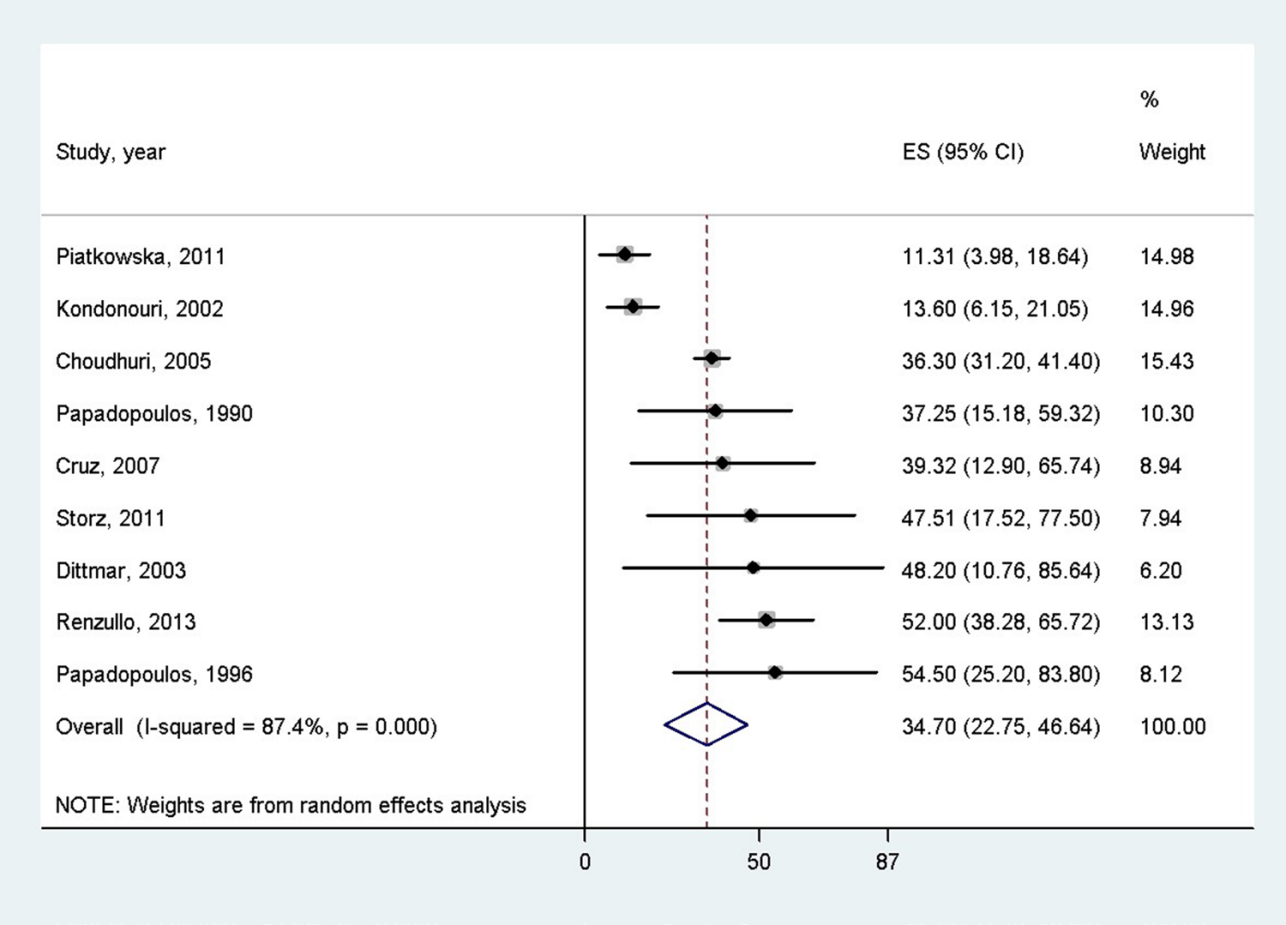
Mean age of polyglandular autoimmune syndrome patients; the pooled mean age of polyglandular autoimmune syndrome patients was 34.7 years

**Fig. 3:**
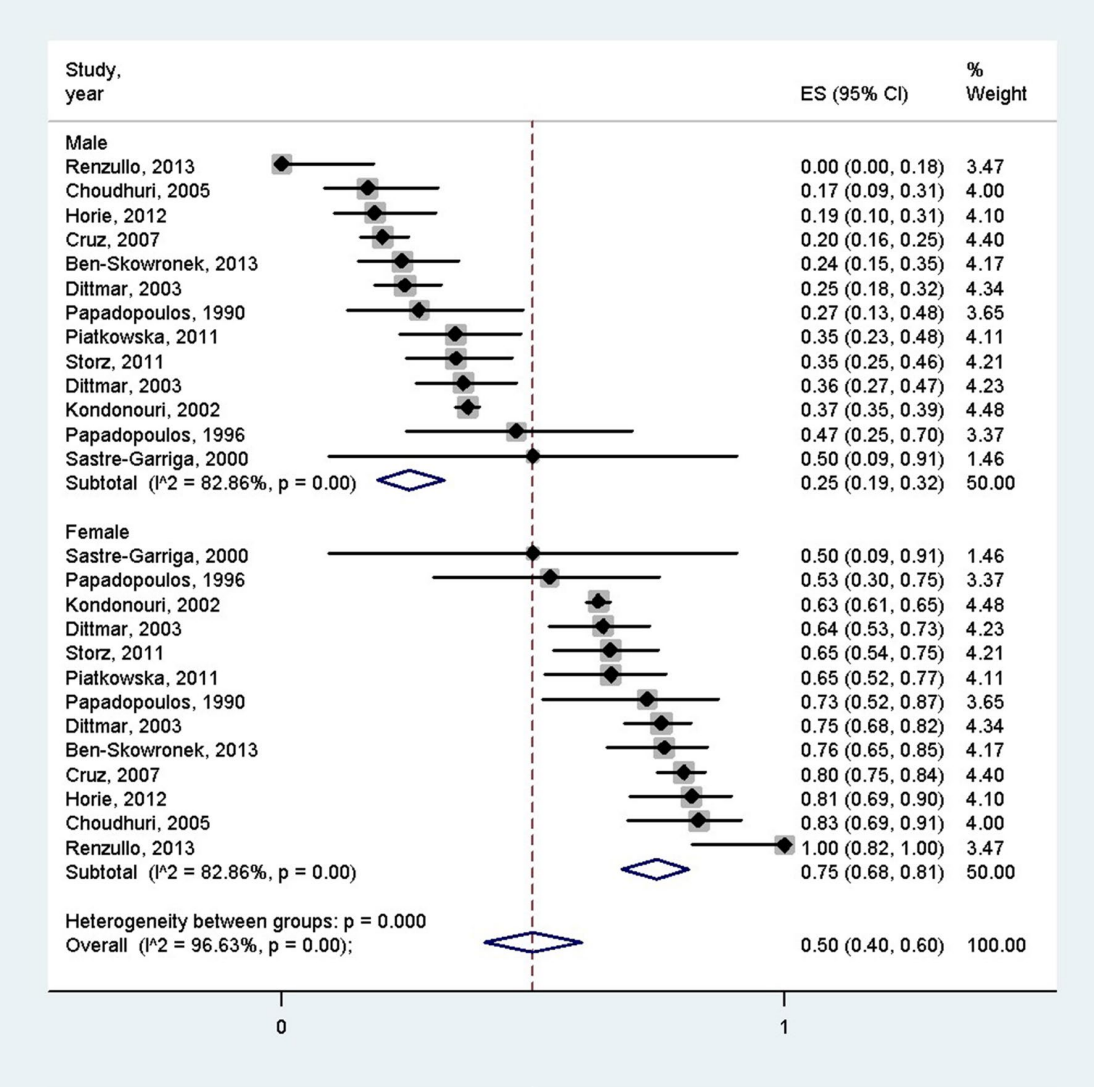
Gender distribution of polyglandular autoimmune syndrome patients; females were more often affected by polyglandular autoimmune syndrome than males

**Fig. 4:**
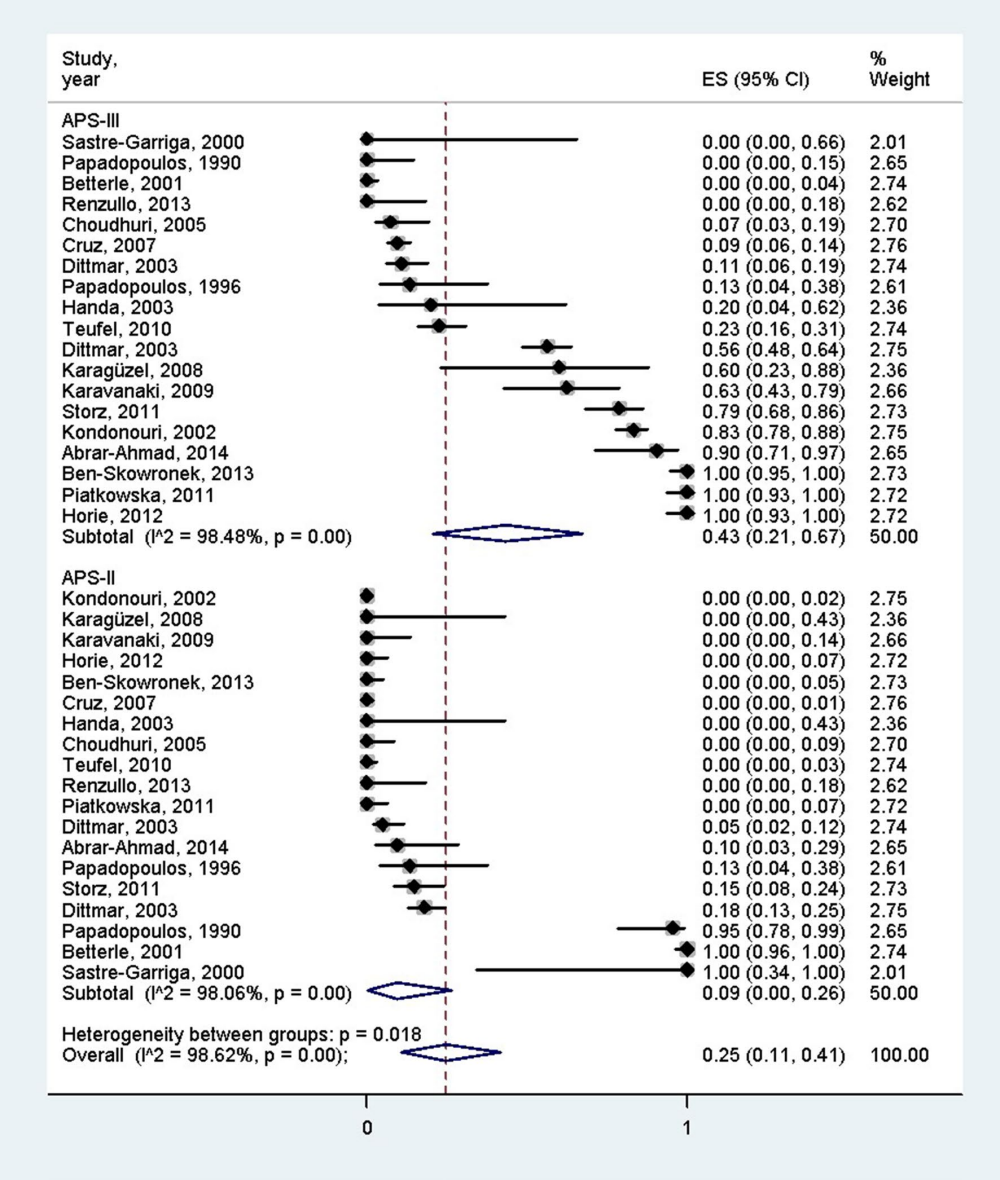
Distribution of patients between polyglandular autoimmune syndrome type II and III; the proportion of polyglandular autoimmune syndrome type III patients was significantly higher than that of polyglandular autoimmune syndrome type II patients

According to 9 studies which mentioned the age of the patients, the overall mean age of the PAS patients was 34.7 years (95% CI: 22.75–46.64 years) [3, 19–26] (Fig. 2). Females were more often affected by PAS than males (75% [95% CI 68–81%] vs. 25% [95% CI = 19–32%], *p* < 0.001), according to the 12 articles containing the female–male ratio [3, 19–28] (Fig. 3). Distinction between PAS II and PAS III was made in 842 cases, 177 and 665 of which were PAS II and III (21.1% vs 78.9%), respectively [3, 11, 19–24, 26–36]. There was a significant difference between these groups according to the pooled effect sizes (9% (95% CI 0–26%) vs 43% (95% CI 21–67%) (Fig. 4). The most prevalent autoimmune endocrinopathies were AITDs, T1DM and AD (970, 697 and 174 cases, respectively).

### Prevalence of other autoimmune disorders beyond the obligatory manifestations of PAS II and PAS III

In PAS II, AD coexisted with AITDs, T1DM or the combination of these conditions in 114, 32 and 18 cases, respectively. In addition, one other endocrine (POF) and five non-endocrine organ-specific autoimmune disorders (pernicious anemia, alopecia, coeliac disease, multiple sclerosis and vitiligo) were reported.

In PAS III, two other autoimmune endocrinopathies (POF and T1DM), six non-endocrine organ-specific (vitiligo, autoimmune hepatitis, pernicious anemia, myasthenia gravis, coeliac disease and alopecia) and four systemic autoimmune disorders (RA, SLE, Sjögren’s syndrome and psoriasis) were detected in combination with AITDs [3, 11, 19–24, 26–35].

### The number of associated autoimmune disorders in PAS II and III

**Fig. 5:**
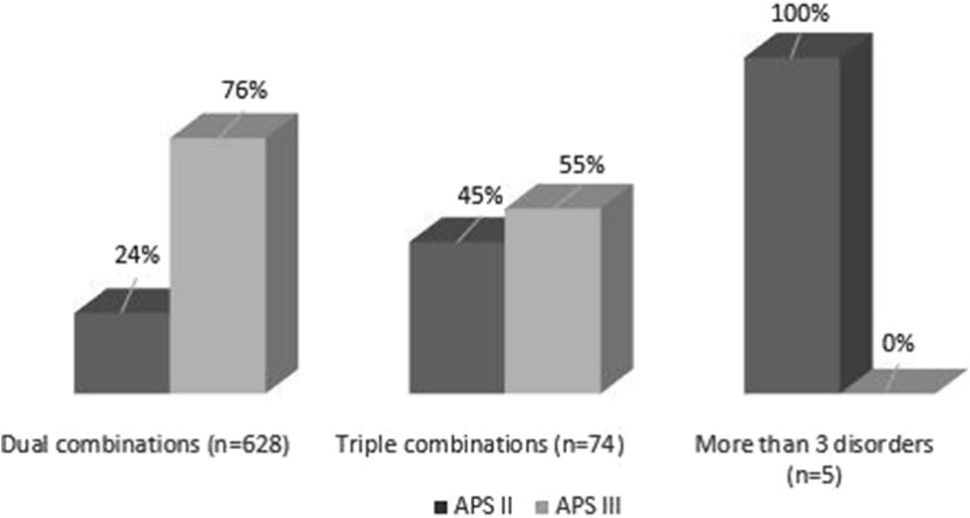
Distribution of the combinations of two, three and more than three disorders among polyglandular autoimmune syndrome type II and type III patients

The patients were separated into three subgroups as follows: patients with a combination of two, three and more than three disorders. The proportion of dual combinations among PAS III patients was significantly higher than in PAS II patients [476 (75.7%) vs 152 (24.3%), *p* < 0.001]. There was no significant difference in the frequencies of triple combinations between the PAS II and PAS III patients (33 (44.6%) vs 41 (55.4%), *p* = 0.739), while patients with more than three autoimmune disorders were only found in PAS II (5 (100.0%) vs 0 (0.0%), *p* = 0.001) (Fig. 5).

### Combinations of autoimmune disorders with AD

Among PAS II, 174 combinations were diagnosed. AD occurred in dual combinations in 83.9% of the PAS II patients. Among these patients, only AITD and T1DM were found in dual combinations in 114 and 32 cases, respectively. Triple combinations were diagnosed in 18 cases (10.3%), only 13 of which (7.5%) showed the classical triad of PAS II (AD, AITD and T1DM). Beyond these conditions, alopecia, POF, coeliac disease, pernicious anemia, multiple sclerosis and vitiligo developed with AD.

Autoimmune bowel diseases, autoimmune hepatitis, hypoparathyroidism, hypophysitis, myasthenia gravis, psoriasis, rheumatoid arthritis, Sjögren’s syndrome and SLE were not reported in combination with AD. Interestingly, systemic autoimmune disorders coexisted with AD only if four or five autoimmune disorders developed.

## Discussion

PAS is a complex and heterogeneous disorder. The fact that these conditions are so rare and that the number of variations is so high make the use of evidence-based approach difficult [1, 7]. The screening and diagnostic protocol of these patients is a hard task for clinicians [34]. To our knowledge, this is the first meta-analysis in the field of PAS.

In PAS II, AD is present in 100% of the cases, AITDs in 69–82% and T1DM in 30–52% of the patients [36]. No analysis of the other coexisting autoimmune disorders was previously available. The aim of this meta-analysis was to identify the main characteristics of PAS patients according to age, gender and combination of autoimmune disorders to develop relevant diagnostic and screening protocols. PAS II frequently appears later than PAS I, mostly in young adulthood [37]. The mean age of the PAS patients at the time of diagnosis in our work was 34.7 years, which is unexpectedly high [38, 39]. It is well known that PAS are more common in females [1, 12] and this was confirmed by our study as well. As opposed to the common belief, PAS III was more prevalent than PAS II [25]. This is due to the much higher prevalence of AITDs in comparison with AD, which is a diagnostic criteria for PAS II [12]. In fact, AITDs are the most common autoimmune endocrinopathies in combination with other autoimmune conditions [13]. According to literature data, dual combinations in PAS II are more common than the classical triad of AD, AITDs and T1DM, which appears true only for approximately 10–20% of cases [40]. According to our results, AD occurred in dual combinations in 83.9% of the PAS II patients, while the proportion of the triple combinations was 10.3%. The combination of AD, AITD and T1DM was diagnosed in 7.5% of PAS II patients. More than three autoimmune manifestations are more common in patients who have Addison’s disease [37]. Unfortunately, the categorization of the patients suffering from two or more autoimmune disorders in the PAS subgroups is not a clear task for the clinicians [1, 12]. These patients are receiving medications and therapies for their disorders separately and their conditions are not managed as a part of a complex disease. This may be the reason why only 18 articles fulfilled the inclusion criteria in this meta-analysis.

There are some limitations of our meta-analysis. We found case studies in a large number, while case series—studies analyzing larger populations—were found in a limited number. The data found in case studies could not be used for statistical analyses. In the future, the classification of PAS patients suffering from two or more autoimmune disorders is important to better understand the epidemiological data and the possible combinations of autoimmune disorders. The prevalence of autoimmune thyroid diseases is 40–200 times higher than that of Addison’s disease in the general population and in association studies, the reference disorder must be the most frequent. Development of consecutive registries for autoimmune thyroid disorders and other autoimmune endocrinopathies seems to be essential to estimate the real prevalence of co-associations.

PAS II and PAS III are both due to polymorphisms in the HLA DQ/DR regions [10]. PAS II has been found to be strongly associated with HLA haplotypes with DR3/DQ2 and DR4/DQ8 and with DRB1*0404 [10]. Identification of the affected regions may be useful to estimate the risk of PAS. Circulating organ- and cell-specific autoantibodies can be detected in patients with the syndrome [41]. However, it is difficult and expensive to search for all these markers during the follow-up period. Based on risk assessment, the screening of high-risk individuals would be possible. The screening process is further complicated by the late manifestation of the second autoimmune disorder; decades may elapse between two autoimmune manifestations [42]. Detailed data are not available in the literature, few reports found that the shortest interval may be between AD and AITD, the longest between T1DM or vitiligo and AITDs [42]. There are some studies about the prevalence of combined autoimmunities among children and adults [14]. Association of autoimmune disorders is more common in adult patients; however, many autoimmune diseases can develop in childhood [14].

In conclusion, our meta-analysis clearly confirmed that the association of various other forms of autoimmune disorders which are not the obligatory components of PAS is not uncommon. However, AITDs, T1DM and AD are the most frequent combinations occurring in PAS, thus screening for these conditions seems to be reasonable. The development of relevant diagnostic and screening protocols to identify these patients timely is warranted.

## Electronic supplementary material

Below is the link to the electronic supplementary material.

Supplementary file1 (PDF 178 kb)
