# Supplementary material for: Prevalence of other autoimmune diseases in polyglandular autoimmune syndromes type II and III
Source: J Endocrinol Invest. 2020 Aug 17;43(9):1–9. doi: 10.1007/s40618-020-01229-1 (PMC7431444; doi:10.1007/s40618-020-01229-1)
Supplement: Supplementary file 1 — Supplementary file1 (PDF 178 kb) [file 40618_2020_1229_MOESM1_ESM.pdf]

		Is the objective of the study stated clearly in the abstract, introduction, or methods section?	Are the characteristics of the participants included in the study described?	Were the cases collected in more than one centre?	Are the eligibility criteria (inclusion and exclusion criteria) for entry into the study explicit and appropriate?	Were participants recruited consecutively?	Did participants enter the study at a similar point in the disease?	Is the cases described with sufficient details to allow other investigators to replicate the research or to allow practitioners make inferences related to their own practice?
Abrar-Ahmad	2014	*	*	*	*	*		
Ben-Skowronek	2013	*	*		*	*	*	*
Betterle	2001	*	*		*	*	*	*
Choudhuri	2005		*		*	*		
Cruz	2007	*	*		*	*	*	*
Dittmar	2003	*	*		*	*		*
Handa	2003	*	*			*	*	*
Horie	2012	*	*		*		*	*
Karagüzel	2008	*	*		*	*		*
Karavanaki	2009	*	*		*	*	*	*
Kondonouri	2002	*	*	*	*	*		
Papadopoulos	1990	*	*		*	*	*	*
Papadopoulos	1996	*	*	*	*	*	*	
Piatkowska	2011	*	*			*		*
Renzullo	2013		*		*	*	*	*
Sastre-Garriga	2000	*			*	*	*	
Storz	2011	*	*	*	*	*		*
Teufel	2010	*	*		*	*	*	*

Supplementary table. 1. Quality assesment of the included studies

First Author	Year of publication	Design	Country	Number of patients		Age in APS			Sex in APS	
				APS	not APS	Mean	SD	range	Female	Male
Abrar-Ahmad	2014	Case series	France	21	7	NA	NA	NA	NA	NA
Ben-Skowronek	2013	Case series	Poland	67	394	NA	NA	NA	51	16
Betterle	2001	Retrospective review	Italy	100	147	NA	NA	NA	NA	NA
Choudhuri	2005	Retrospective review	India	41	0	36,3	2,6	7-68	34	7
Cruz	2007	Retrospective review	Brazil	254	0	39,32	13,48	NA	203	51
Dittmar	2003	Retrospective review	Germany	151	0	NA	NA	NA	114	37
				83	388	48,2	19,1	NA	53	30
Handa	2003	Retrospective review	India	5	620	NA	NA	NA	NA	NA
Horie	2012	Retrospective review	Japan	54	143	NA	NA	NA	44	10
Karagüzel	2008	Cohort study	Turkey	5	52	NA	NA	NA	NA	NA
Karavanaki	2009	Retrospective review	Greece	24	120	NA	NA	NA	NA	NA
Kondonouri	2002	Retrospective review	Germany and Austria	210	17539	13,6	3,8	NA	NA	NA
Papadopoulos	1990	Retrospective review	Sweden	22	22	35	NA	18-61	16	6
Papadopoulos	1996	Retrospective review	Sweden	15	63	57	NA	26-78	8	7
Piatkowska	2011	Retrospective review	Poland	55	327	11,31	3,74	NA	36	19
Renzullo	2013	Retrospective review	Italy	17	98	52	7	NA	17	0
Sastre-Garriga	2000	Retrospective review	Spain	2	1275	NA	NA	28-37	1	1
Storz	2011	Retrospective review	Germany	75	75	47,51	15,3	NA	49	26
Teufel	2010	Retrospective review	Germany	111	167	NA	NA	NA	NA	NA

Table 2. Characteristics of the included studies (NA= not available)
